# When West Meets East: The Origins and Spread of Weedy Rice Between Continental and Island Southeast Asia

**DOI:** 10.1534/g3.119.400021

**Published:** 2019-07-10

**Authors:** Ting Xiang Neik, Jing-Yun Chai, Seow-Yeen Tan, Maggie Pui San Sudo, Yongxia Cui, Jayasyaliny Jayaraj, Su-Sin Teo, Kenneth M. Olsen, Beng-Kah Song

**Affiliations:** * School of Science, Monash University Malaysia, 46150 Bandar Sunway, Selangor, Malaysia; † School of Biological Sciences, University of Western Australia, Perth, Australia; ‡ College of Science, Sichuan Agriculture University, Yaan 625014, Sichuan, China; § Department of Agriculture, Sabah, Malaysia; ** Washington University in St Louis, Department of Biology, St. Louis, MO 63130, and; †† Monash University Malaysia Genomics Facility, Tropical Medicine and Biology Multidisciplinary Platform, 47500 Bandar Sunway, Selangor, Malaysia

**Keywords:** adaptive evolution, agricultural weeds, awn length, crop-weed introgression, *Oryza sativa*, weedy rice

## Abstract

Weedy crop relatives are among the world’s most problematic agricultural weeds, and their ability to rapidly evolve can be enhanced by gene flow from both domesticated crop varieties and wild crop progenitor species. In this study, we examined the role of modern commercial crop cultivars, traditional landraces, and wild relatives in the recent emergence and proliferation of weedy rice in East Malaysia on the island of Borneo. This region of Malaysia is separated from the Asian continent by the South China Sea, and weedy rice has become a major problem there more recently than on the Malaysian peninsular mainland. Using 24 polymorphic SSR loci and genotype data from the awn-length domestication gene *An-1*, we assessed the genetic diversity, population structure and potential origins of East Malaysian weeds; 564 weedy, cultivated and wild rice accessions were analyzed from samples collected in East Malaysia, Peninsular Malaysia and neighboring countries. While there is considerable evidence for contributions of Peninsular Malaysian weed ecotypes to East Malaysian populations, we find that local crop cultivars and/or landraces from neighboring countries are also likely contributors to the weedy rice infestations. These findings highlight the implications of genetic admixture from different cultivar source populations in the spread of weedy crop relatives and the urgent need for preventive measurements to maintain sustainable crop yields.

Agricultural weeds are a primary constraint on crop productivity in many agricultural areas worldwide. Competition from agricultural weeds can result in yield losses exceeding 80% (Diarra *et al.* 1985), making them a primary threat to global food security (Savary *et al.* 2012; Soltani *et al.* 2016). The emergence and spread of agricultural weeds is the result of multiple interacting factors; these include non-regulated farming methods that reflect local cultural practices, adoption of the no-tillage and direct-seeding methods that are associated with modern-day mechanized farming, and sharing of farm equipment and irrigation networks that facilitate the spread of weed infestations (Barroso *et al.* 2006; Calha *et al.* 2014; Ghosh *et al.* 2017).

Some of the most problematic agricultural weeds are close relatives of domesticated species (Ellstrand *et al.* 2010). These weedy crop relatives are typically characterized by a combination of crop-like traits (*e.g.*, erect growth architecture, annual life history) and wild-like traits (*e.g.*, seed shattering, seed dormancy) which together make them highly adapted to invade agroecosystems (Vigueira *et al.* 2013). Ongoing hybridization with crop varieties and/or wild progenitor populations can further promote their proliferation (Ellstrand *et al.* 2013; Harlan 1992). Indeed, gene flow between cultivated, weedy and wild populations can occur spontaneously under cultivated and/or wild settings and is considered the main driving force behind the establishment of weediness traits in weedy crop relatives (Lu *et al.* 2016; Sultan *et al.* 2013; Warschefsky *et al.* 2014). Understanding the mechanisms by which weedy crop relatives evolve and the role of gene flow from cultivated and/or wild populations in their evolution can provide important insights for devising effective weed control strategies.

The weedy conspecific form of cultivated rice (*Oryza sativa* L.) has become an increasing threat to rice farming worldwide in recent decades (Chauhan 2013). Notorious for its easily-shattering seeds and ability to aggressively outcompete crop varieties for light and nutrients, weedy rice shows a striking morphological resemblance to cultivated rice in its vegetative growth, which hinders detection and eradication of the weed in the field. If left uncontrolled, weedy rice infestations can reduce harvests by more than 90% (Singh *et al.* 2013). Morphologically, weedy rice strains vary widely in grain phenotypes and range from those that closely resemble cultivated rice (referred to as strawhull awnless or SH forms) to those resembling wild *Oryza* species (blackhull awned or BHA forms) (*e.g.*, Song *et al.* 2014). Studies of weedy rice populations in regions where the rice wild progenitor (*Oryza rufipogon*) can be found growing near rice fields (Southeast Asia, southern China, South Asia) have indicated an important role of hybridization between wild and cultivated populations in the origin and evolution of local weedy rice populations (Huang *et al.* 2017; Pusadee *et al.* 2013; Song *et al.* 2014; Vigueira *et al.* 2019; Zhang *et al.* 2012). Although both cultivated and weedy rice are predominantly self-pollinating, outcrossing occurs at low frequencies (∼1%) (Cao *et al.* 2006; Gealy *et al.* 2003). This can result in hybridization and migration of adaptive alleles into weed populations, including those derived from crop varieties (*e.g.*, herbicide resistance; Burgos *et al.* 2014), as well as those from wild populations (*e.g.*, seed shattering and dormancy; Cui *et al.* 2016). In Southeast Asia, both crop-to-weed and wild-to-weed allelic introgression has been documented in Thailand (Pusadee *et al.* 2013; Wedger *et al.* 2019) and Malaysia (Cui *et al.* 2016; Sudianto *et al.* 2016), indicating that continuous outcrossing and introgression into weedy rice populations can contribute to their adaptation and proliferation.

In recent decades, shifts away from traditional hand-transplanted rice farming to mechanized direct-seeding of rice fields have led to a rapid rise in weedy rice infestations in Southeast Asia (Chauhan 2013; Pusadee *et al.* 2013; Song *et al.* 2014; Vigueira *et al.* 2019). Within this region, Malaysia presents an especially dynamic history of weedy rice emergence and evolution. This country comprises two distinct geographical regions separated by the South China Sea (Figure 1), and these have experienced different histories of weedy rice proliferation. In Peninsular Malaysia (PM; also known as West Malaysia), located on the Asian continent, weedy rice was first reported in the late 1980s where it soon became a major weed pest (Wahab and Suhaimi 1991). Yield losses ranging from 60 to 100% have been recorded there (Azmi and Karim 2008). Previous studies of Peninsular Malaysian weedy rice revealed an important role of modern elite Malaysian cultivars (specifically, the ‘Malaysian Rice’ MR) series developed by the Malaysian Agricultural Research & Development Institute (MARDI), as well as local wild rice populations, in the origin and proliferation of the weeds (Cui *et al.* 2016; Song *et al.* 2014; Sudianto *et al.* 2016; Vigueira *et al.* 2019).

**Figure 1:**
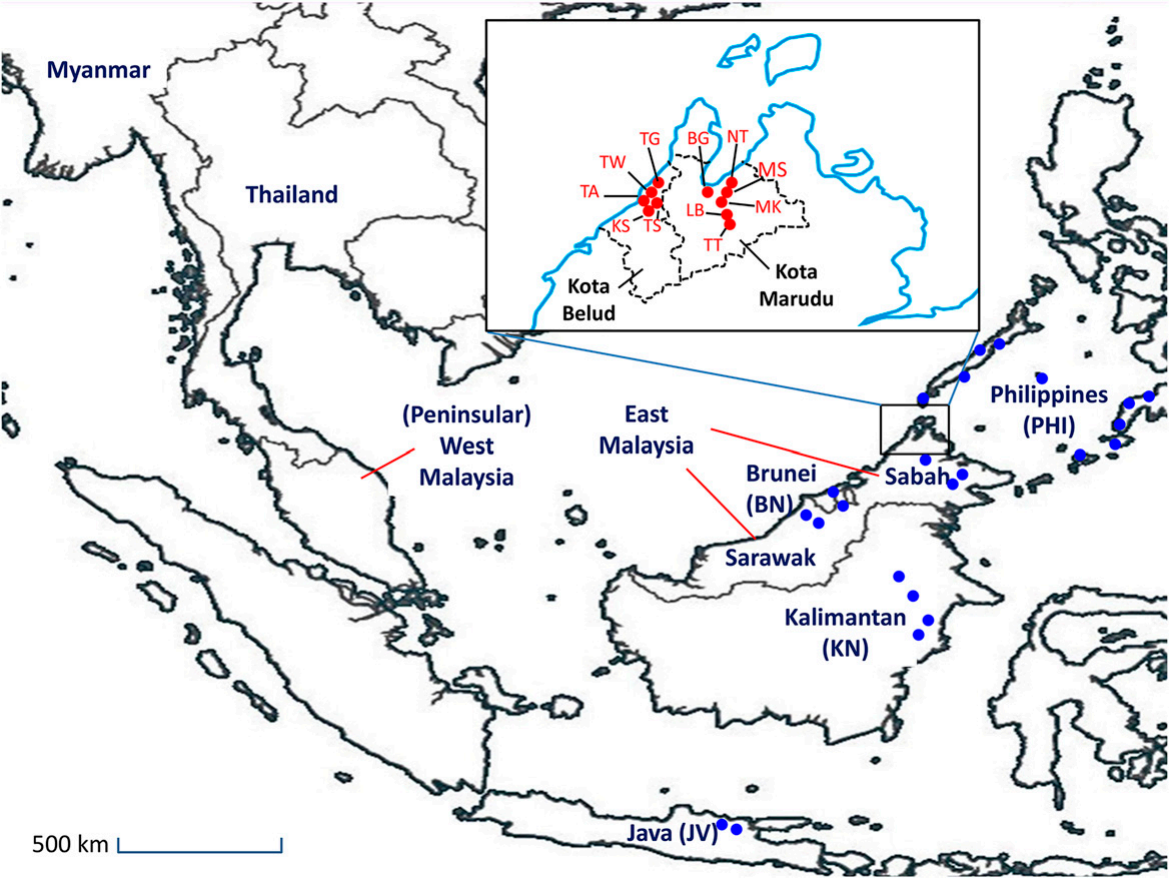
Map of Sabah and surrounding regions showing geographical locations for the 11 weedy rice populations surveyed in this study. The population codes at each collection site represent the name of the villages in the districts Kota Marudu and Kota Belud. Red and blue dots indicate Sabah weedy rice and other landrace rice sampling sites, respectively. Abbreviations used: KS, Kampung Sangkir; TS, Kampung Taun Usik; TW, Kampung Tawadakan; TA, Kampung Tamau; TG, Kampung Timbang; MK, Kapmung Mangkalua; MS, Kampung Masolog; NT, Kampung Nolotan; LB, Kampung Longob; BG, Kampung Balagaton; TT, Kampung Telangtang; MG, Kampung Meniggi.

In contrast to Peninsular Malaysia, the region of East Malaysia, comprising Sabah and Sarawak states on the island of Borneo, was free from major weedy rice infestations until the 2000s. Weedy rice was first observed there in the Kota Belud and Kota Marudu districts of Sabah state (Figure 1) (S. S. Teo, unpublished observations). It has become a widespread problem in Sabah since 2007, with up to 90% yield loss reported in local rice fields (Bernama 2009), and commensurate impacts on farmer income. Notably, unlike Peninsular Malaysia, East Malaysia does not have wild rice populations present. In addition, the modern cultivars of this region differ from those of Peninsular Malaysia, being characterized by higher levels of phenotypic and genetic variation and by little use of the elite MR cultivar series (Ministry of Agriculture Malaysia 1996). Traditional local rice landraces of East Malaysia are also different from those of Peninsular Malaysia (S. S. Teo, unpublished observations). Nonetheless, Sabah weedy rice strains are not phenotypically distinct from those of Peninsular Malaysia, which suggests that they could have originated through accidental introductions from the western part of the country. Alternatively, the close geographical proximity of East Malaysia to other rice-growing countries, including Indonesia and the Philippines, raises the possibility that introductions from those regions could also contribute to the emergence of Sabah weeds.

Here, we examined the genetic composition of weedy rice from Sabah state in East Malaysia to compare its evolution with that of western Peninsular Malaysia weedy rice. Taking advantage of a previously published Simple Sequence Repeat (SSR) dataset for Peninsular Malaysia weeds (Song *et al.* 2014), we genotyped Sabah weeds using the same set of 24 loci for a combined analysis. We also sampled and genotyped Sabah rice landraces and local high-yielding cultivars and rice germplasm from elsewhere in East Malaysia (Sarawak state), as well as rice varieties from Indonesia (Java and Kalimantan), Brunei, and the Philippines, to assess their potential role in the Sabah weeds’ evolution. As a complementary approach to neutral marker analyses, we examined allelic variation at a well-characterized rice domestication gene, *Awn-1* (*An-1*), which encodes a basic helix-loop-helix (bHLH) protein and controls the development of the long awns that are characteristics of wild rice and some weedy rice strains (Cui *et al.* 2016; Luo *et al.* 2013; Qi *et al.* 2015). Domesticated rice underwent widespread selection for loss-of-function alleles at *An-1*, and haplotype information at this locus can provide insights into patterns of introgression between cultivars, weedy and wild rice (Cui *et al.* 2016). Our aim in this study was to address the following specific questions: (1) What role have local Sabah cultivars and/or landraces played in the evolution of Sabah weedy rice? (2) What role have Peninsular Malaysian weed strains played in the establishment of these East Malaysian weed populations? (3) Given the close geographical proximity of East Malaysia to other countries, including Indonesia, Philippines, and Brunei, what role, if any, has rice from those regions played in the evolution of Sabah weedy rice? (4) What does *An-1* haplotype variation reveal about the origin of Sabah weedy rice and the molecular basis of awn length variation in these weeds?

## Materials and Methods

### Plant material

A total of 138 seed samples representing 11 populations of Sabah weedy rice were collected in 2011 (Figure 1; Table S1, Supporting information). These accessions cover approximately 1,600 ha of rice planting areas in Kota Marudu and Kota Belud, which are the two major rice planting districts covering about one-third of the rice fields in the state. One mature panicle per plant was harvested and regarded as one accession. Phenotypically, the Sabah weedy rice collection showed a gradation in grain characteristics, and accessions were classified as follows: 50 straw-hull awnless (SbSH), 15 strawhull awned (SbSHA), 61 brown-stripe-hull awnless (SbBR), and 12 brown-stripe-hull awned (SbBRA) types (Figure S1, Tables S2 and S3, Supporting information).

In addition to weedy rice accessions, sampling also included cultivated rice varieties (both modern cultivars and traditional landraces) from Sabah and neighboring regions. Within Sabah, 15 high-yielding Sabah cultivars (SbCV) were collected from different rice planting areas. Here, we use the term ‘high-yielding cultivars’ to refer to the group of Sabah rice varieties that have been improved using modern breeding techniques for use in large-scale commercial rice production. These include commonly planted varieties developed by the local agricultural agencies or introduced from neighboring regions by immigrants. In contrast, ‘modern elite cultivars’ in this study exclusively refers to cultivars developed by Malaysian Agricultural Research and Development Institute (MARDI) and named in the ‘Malaysian Rice’ (MR) series; those cultivars have been widely planted in Peninsular Malaysia but not East Malaysia over the past three decades. The Sabah landrace group (abbreviated SbLr) was represented by 27 accessions obtained from the International Rice Research Institute (IRRI) (Table 1); landraces are defined as cultivated varieties that are mostly no longer planted by modern day farmers in large scale operations (Harlan 1992). IRRI landraces were also sampled from Sarawak state in East Malaysia (15 accessions), and from the following neighboring countries: Philippines (20 accessions), Indonesia (32 accessions from Kalimantan, 9 accessions from Java) and Brunei (10 accessions) (Table 1). Sampling was further supplemented with 19 and 29 *Oryza rufipogon* accessions respectively sampled from Peninsular Malaysia and other Southeast Asian countries. One plant per accession was grown in the plant house of Monash University Malaysia for DNA extraction.

**Table 1:** Genetic diversity in Sabah and Peninsular Malaysia weedy rice samples grouped by morphotype. Abbreviations of genetic diversity parameters are as follows: N_a_, number of accessions; P, percentage of polymorphic loci; *H*_o_, observed heterozygosity; *H*_e_, expected heterozygosity; M_A_, number of morphotype-specific (private) alleles; *I*, Shannon diversity index; *Fis*, inbreeding coefficient. Data for Peninsular Malaysia samples are derived from Song *et al.* (2014)

Morphotype code	N	*P (%)*	*H*_o_	*H*_e_	M_A_	*I*	*Fis*
***Sabah weedy and cultivated rice:***							
SbSH	50	95.8	0.030	0.534	21	1.080	0.946
SbSHA	15	91.7	0.036	0.335	0	0.634	0.899
SbBR	61	95.8	0.047	0.478	12	0.952	0.904
SbBRA	12	91.7	0.031	0.476	14	0.862	0.940
All Sabah weedy rice	138	93.8	0.036	0.456		0.882	0.927
Sabah cultivars (SbCV)	15	87.5	0.031	0.479	6	0.873	0.940
Sabah landraces (SbLr)	27	95.8	0.011	0.620	34	1.249	0.944
***Peninsular Malaysian weedy rice (from*** ***Song et al. 2014******):***							
PMSH	36	83.3	0.097	0.231	1	0.468	0.604
PMSHA	17	87.5	0.087	0.332	3	0.602	0.754
PMmSH	27	95.8	0.120	0.413	2	0.784	0.720
PMmSHA	15	87.5	0.064	0.392	1	0.703	0.848
PMBR	68	95.8	0.031	0.310	18	0.620	0.902
PMBRA	28	91.6	0.037	0.384	6	0.761	0.904
PMBH	5	41.6	0.033	0.247	3	0.434	0.893
PMBHA	10	79.1	0.021	0.286	6	0.497	0.937
All PM weedy rice	206	77.5	0.065	0.368		0.647	0.853
***Peninsular Malaysian cultivated and wild rice:***							
Elite cultivar (PMCV)	25	70.8	0.033	0.085	4	0.169	0.621
*O. rufipogon* (PMOr)	18	100.0	0.332	0.738	58	1.599	0.595

Abbreviations of rice groups: SbSH, Sabah strawhull awnless weeds; SbSHA, Sabah strawhull awned weeds; SbBR, Sabah brown-striped hull, awnless weeds; SbBRA, Sabah brown-striped hull, awned weeds; SbCV, Sabah *O. sativa* high-yielding cultivar; PMCV, PM *O. sativa* modern elite cultivar; SbLr, Sabah landrace rice; PMSH, strawhull awnless PM weeds; PMSHA, strawhull awned PM weeds; PMBR, brown-striped hull, awnless PM weeds; PMBRA, brown-striped hull, awned PM weeds; PMBH, blackhull awnless PM weeds; PMBHA, blackhull awned PM weeds; PMmSH, morphologically intermediate weed form between SH and BR; PMmSHA, morphologically intermediate weed form between SHA and BRA; PMOr, *O. rufipogon* accessions collected from Malaysia.

### DNA extraction and SSR genotyping

Total genomic DNA was extracted from young healthy leaf tissue using Qiagen DNeasy Plant Mini kits (QIAGEN, Valencia, CA). A panel of 24 SSR loci was used, following Song *et al.* (2014). PCR amplifications were performed in a total of 8 µL reactions containing 20 ng of template DNA, 20 mM Tris-HCl (pH 8.0), 0.2 µM of each primer, 0.2 mM of each dNTPs, 2 mM MgCl_2_ and 0.2 unit of *Taq* polymerase (Platinum *Taq*, Invitrogen). The forward primers were labeled with 6FAM, HEX or NED fluorescent dye. Multiplexing PCR amplification was performed as described previously in Song *et al.* (2014). Amplified products were electrophoresed on an ABI 3130xl genetic analyzer (Applied Biosystems, Foster City, CA, USA) in the Forest Research Institute Malaysia (FRIM). Six rice accessions (MR220, MR211, SH-SBTG02, SHA-SBTS09, BR-SBTA03, and BRA-SBTA25) which were genotyped alongside samples used in Song *et al.* (2014), were included in all genotyping analyses. These reference standards were used for genotyping calibration to ensure consistency in assignment of allele sizes across Sabah and Peninsular Malaysia samples. SSR allele sizes were binned and scored, followed by manual checking and correction of the scored alleles with the aid of GeneMarker 2.6.0 (SoftGenetics, State College, PA, USA).

### Genetic analysis

Measures of Sabah weedy rice genetic diversity, including the allele number per sampling location (N_a_), percentage of polymorphic loci (*P*), observed heterozygosity (*H*_o_), expected heterozygosity (*H*_e_), Shannon’s diversity index (*I*) and inbreeding coefficient (*F*_is_), were calculated using PowerMarker V3.25 (Liu and Muse 2005). Significant differences between and within population samples across all microsatellite markers were evaluated using Wilcoxon signed rank test in XLSTAT version 2019.2.1.58717. C.S. Chord genetic distance values Cavalli-Sforza and Edwards (1967) were obtained using PowerMarker V3.25 (Liu and Muse 2005). Comparative analyses included 279 previously-genotyped samples from Song *et al.* (2014), consisting of 206 Peninsular Malaysian weedy rice accessions, 25 Peninsular Malaysia MR cultivars (PMCV), 19 *tropical japonica* US crop varieties, 18 Malaysian wild rice accessions (PMOr) and 11 wild rice accessions representing other Southeast Asian (SEA) countries (Thailand, Cambodia and Myanmar; SEAOr). Weed accessions were assigned morphotype-based categories in a dataset that merged Sabah genotype data with the previously analyzed Peninsular Malaysia accessions; the Peninsular Malaysia accessions have been previously classified into 8 morphotype groups: strawhull awnless (PMSH, 36 samples), strawhull-awned (PMSHA, 17 samples), intermediate strawhull awnless (PMmSH, 27 samples), intermediate strawhull-awned (PMmSHA, 15 samples), brownhull awnless (PMBR, 68 samples), brownhull-awned (PMBRA, 28 samples), blackhull awnless (PMBH, 5 samples) and blackhull-awned (PMBHA, 10 samples).

A combined data set of 564 rice accessions, comprising 430 Malaysian rice accessions (including 344 weedy, 19 wild, 40 cultivated and 27 landrace rice), 86 landrace accessions from neighboring countries, 19 *tropical japonica* rice varieties from the USA, and 29 Southeast Asian wild rice accessions, were subjected to population structure analysis using model-based Bayesian-clustering program STRUCTURE ver 2.3.3 (Pritchard *et al.* 2000). An admixture model was run five times for each value of *K* assumed subpopulations (with K ranging from 1 to 17), using 200,000 iterations after a burn-in of 100,000 iterations. The Δ*K* ad hoc statistic of Evanno *et al.* (2005) was used to assess the K value that best approximates population structure. Optimal K was further assessed based on consistency of membership assignments over replicate runs at a given K value. Results were visualized in DISTRUCT (Rosenberg 2003). As a complement to STRUCTURE analyses, a PCoA clustering analysis was implemented in the software GeneAlEx 6 (Peakall and Smouse 2006) using pairwise C.S. chord genetic distance values between rice accessions (Cavalli-Sforza and Edwards 1967). Grouping of the rice samples based on eigenvalues was performed to summarize and condense the variance among individuals to a limited number of dimensions, allowing for identification of genetically similar clusters.

### Allelic variation at An-1

A total of 181 rice accessions comprising 63 Peninsular Malaysia weedy rice, 53 Sabah weedy rice, 10 Peninsular Malaysia cultivars, 5 Sabah cultivated rice, 10 Sabah landraces, 22 neighboring regions’ landraces (comprising 14, 3, 3, and 2 landraces respectively from Kalimantan (Indonesian Borneo), the Philippines, Java, and Brunei), and 18 wild rice accessions were genotyped for *An-1* allelic variation. PCR primers were designed to amplify and sequence a 566 bp region corresponding to exons 1 and 2 which contains three major functional nucleotide polymorphisms identified by Luo *et al.* (2013): GCC/-, C/G, and G/- (forward primer An-01F, 5′-AGCGCCAACAACTCCTGCTAC-3′; reverse primer An-01R, 5′-GCTTCATCCTCTCGCTTATCCTC-3′). PCR amplification was performed in 20 μL reactions containing the following: 20 mM of Tris-HCl (pH 8.0), 50 mM of KCl, 1.5 mM of MgCl_2_, 0.25 μM of each primer, 1M betaine, 1 mM dNTP mix, 0.5 U of Platinum *Taq* DNA polymerase (Invitrogen) and 20 ng of genomic DNA. DNA amplifications were carried out with an initial denaturation at 94° for 5 min, followed by 35 cycles of denaturation at 94° for 30 s, primer annealing at 60° for 30 s and primer extension at 72° for 1 min, with a final extension at 72° for 10 min. Amplified products were sequenced directly with forward and reverse primers using Sanger sequencing (ABI PRISM BigDye Terminator Cycle Sequencing Reaction Kit, Perkin Elmer, USA) at the First BASE Laboratories Sdn. Bhd. (Malaysia) and Washington University Biology Departmental core facility. Reference *An-1* haplotypes were obtained from published sequences of awned wild rice *O. rufipogon* (W1943), the awnless *indica* cultivar HP228 and the reference *japonica* cultivar Nipponbare (Luo *et al.* 2013), for comparison with the sequences obtained from the present study. All sequences were aligned and checked for SNPs using Sequencher (v4.8, Gene Codes Corp., Ann Arbor, MI). A haplotype network with MP option was constructed using Network 5.0 (Bandelt *et al.* 1999) based on the haplotype information retrieved from this study. Observation that most weedy rice strains carry *An-1* haplotypes of a particular cultivated or wild rice group would suggest that the group has played a major role in the weed’s ancestry.

### Data availability

Newly generated DNA sequences are available in GenBank (MK850861 - MK850929, MK867846 - MK867924, MK867926 - MK867958). Supplemental files available at FigShare, including SSR genotypes saved as a separate file. File Table S1 contains population code, location, number of sample, range of coordinates, coexisting rice varieties of the weedy rice populations and cultivated rice samples used in this study. File Table S2 contains distribution, morphotype code and number of sample of weedy rice accessions used according to hull color and awn presence. File Table S3 contains *Oryza* accession information, haplotype of the *An-1* gene, and coefficents of ancestry inferred by STRUCTURE. File Table S4 contain genetic diversity in Sabah weedy rice samples grouped by sampling location. File Table S5 contains pairwise population F_ST_ values generated by location-based clustering. File Table S6 contains compilation of the ten mutations identified in the sequenced region of *An-1* gene. File Figure S1 contains representatives of five groups of weedy and cultivated rice accessions classified according to seed morphological features. File Figure S2 contains STRUCTURE analysis of the Sabah, Peninsular Malaysian, and other countries’ rice samples. File Figure S3 contains PCoA plot of Sabah, Peninsular Malaysia, and worldwide rice samples. File Figure S4 contains a haplotype network for the *An-1* haplotypes identified in this study. Supplemental material available at FigShare: https://doi.org/10.25387/g3.8268167.

## Results

### Genetic diversity

On average, the observed heterozygosities (*H*_o_) were low (0.036) for all the Sabah weedy rice, comparable with that of Sabah cultivars (*H*_o_ = 0.031) and the Peninsular Malaysian elite cultivar (*H*_o_ = 0.033) (Table 1). Sabah landraces recorded the lowest *H*_o_ at 0.011 (Table 1), indicating a high selfing rate. Genetic diversity of weedy rice accessions from East Malaysia (Sabah state) across all microsatellite markers was significantly higher than that of Western Peninsular Malaysian weeds (*H*_e_ = 0.456 and 0.368 respectively, *P* < 0.0001, Wilcoxon signed-rank test), and also higher than most cultivated rice groups in the analysis (Table 1); the exception was Sabah traditional rice landraces (*H*_e_ = 0.620 with *H*_o_ = 0.011, *I* = 1.249), where the high genetic diversity is a reflection of this group containing a combination of varieties from the genetically diverged *indica* and *japonica* subspecies (see population structure results below). Consistent with previous studies (Li *et al.* 2017; Song *et al.* 2014), wild rice accessions showed significantly higher genetic diversity than any cultivated or weedy rice groups (*H*_e_ = 0.738, *P* < 0.0001, with *H*_o_ = 0.332, *I* = 1.599). Among the different morphotypes of Sabah weedy rice, those with grain phenotypes most closely resembling cultivated rice (the strawhull awnless accessions) exhibited the highest level of genetic diversity (Sabah strawhull awnless, SbSH: *H*_e_ = 0.534 with *H*_o_ = 0.030, *I* = 1.080). However, morphotypes were not structured geographically, and genetic diversity of Sabah weed accessions was comparable in all locations sampled (Table S4, Supporting information). The level of genetic diversity detected in the Sabah weedy rice populations was higher than that of US weedy rice populations analyzed with the same SSR markers (*H*_e_ = 0.27; Gealy *et al.* 2009), and were also higher than reported for weeds in northeastern China (*H*_e_ = 0.31; Cao *et al.* 2006); however, their genetic diversity was similar to values reported for weedy rice from Thailand (*H*_e_ = 0.46; Pusadee *et al.* 2013) and northern Italy (*H*_e_ = 0.48; Jiang *et al.* 2012).

Among the cultivated rice samples, the genetic diversity of Sabah high- yielding cultivars was approximately five times higher than that of the Peninsular Malaysia cultivars (Sabah cultivars, SbCV: *H*_e_ = 0.479, PMCV: *H*_e_ = 0.085, *P* < 0.0001). This significant difference likely reflects the broader genetic background (and correspondingly higher phenotypic diversity) that characterizes Sabah cultivars (Ministry of Agriculture Malaysia 1996), as compared to the more homogenous elite ‘MR’ cultivars planted in the peninsular mainland (Song *et al.* 2014). Low observed heterozygosity values for all weedy and cultivated rice groups in the analysis (*H*_o_ = 0.030 to 0.047) are consistent with the high selfing rate for *O. sativa*, as has been observed for weedy rice in Peninsular Malaysia (*H*_o_ = 0.065; Table 1) and the US (*H*_o_ = 0.02; Gealy *et al.* (2009). In contrast, *H*_o_ was an order of magnitude higher for the wild rice (*O. rufipogon*, *H*_o_ = 0.332), a pattern consistent with its outcrossing mating system.

The inbreeding coefficient (*F*_is_) value of the Sabah weedy rice samples was significantly higher compared to the Peninsular Malaysia weedy rice samples (*F*_is_ = 0.927 *vs. F*_is_ = 0.853, *P* < 0.0001; Table 1), indicating high homozygosity and genetic relatedness within the Sabah weedy rice population. Comparing weedy rice and cultivated rice in both regions, the *F*_is_ values were mostly not significantly different in Sabah (weedy rice *F*_is_ = 0.927, cultivated rice *F*_is_ = 0.940, *P* > 0.0001; Table 1) but mostly significantly different in Peninsular Malaysia (weedy rice *F*_is_ = 0.853, cultivated rice *F*_is_ = 0.621, *P* < 0.0001; Table 1). This suggests minimal cultivated-weedy rice gene flow in Sabah.

### Population structure

A global estimation of *F*_ST_ for the set of 11 Sabah weedy rice sampling locales revealed a low level of population differentiation (*F*_ST_ = 0.065, *P* < 0.001). Pairwise genetic differentiation values between the 11 sampled populations ranged from *F*_ST_ = 0.000 to 0.255, with most values less than 0.05 (Table S5). Consistent with these results, a test for geographical isolation-by-distance indicated no significant correlation between pairwise genetic differentiation and geographical distances in the Sabah weedy rice populations (*r* = 0.1068; *P* > 0.10).

**Figure 2:**
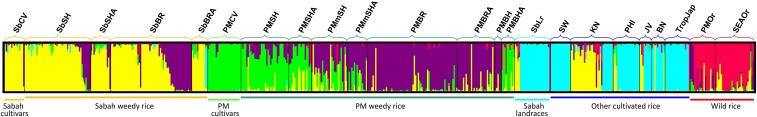
STRUCTURE output at K = 5. Abbreviations used: SbCV, Sabah cultivated rice; PMCV, Peninsular Malaysia cultivated rice; SbSH, Sabah weedy rice with strawhulls and no awns; PMSH, Peninsular Malaysia weedy rice with strawhulls and no awns; PMmSH, Peninsular weedy rice with intermediate strawhulls and no awns; SbSHA, Sabah weedy rice with strawhulls and awns; PMSHA, Peninsular Malaysia weedy rice with strawhulls and awns; PMmSHA, Peninsular Malaysia weedy rice with intermediate strawhulls and awns; SbBR, Sabah weedy rice with brownhulls and no awns; PMBR, Peninsular Malaysia weedy rice with browhulls and no awns; SbBRA, Sabah weedy rice with browhulls and awns; PMBRA, Peninsular Malaysia weedy rice with browhulls and awns; PMBH, Peninsular Malaysia weedy rice with blackhulls and no awns; PMBHA, Peninsular Malaysia weedy rice with blackhulls and awns; PMOr, Peninsular Malaysian *Oryza rufipogon*; PMLr, Malaysian landraces; TropJap, *tropical japonica*; SEAOr, *O. rufipogon* accessions representing other Southeast Asian countries (Thailand, Cambodia and Myanmar).

For the STRUCTURE analysis, evaluation of ΔK (Evanno *et al.* 2005) suggested K = 4 as the best model, with a secondary peak present at K = 10 (Supplementary Figure S2A, B, C). Membership assignments at K = 10 showed little correspondence to known rice subgroups and were not considered further (Figure S2E). At K = 4, population membership assignments were unstable across replicate runs, suggesting that this K-value was also not optimal (Figure S2D) (Gilbert *et al.* 2012). Because a plot of -Ln likelihood values at successive K-values suggested that K = 5 might be a better model than K = 4 for the data (see Supplementary Figure S2A, B, C), we examined multiple run outputs at this K-value. These outputs were stable across replicate runs, and inferred membership assignments for previously-analyzed accessions matched the earlier results (Song *et al.* 2014). We therefore considered K = 5 to be the biologically most realistic population number. Genetic subgroups at K = 5 are shown in Figure 2 and correspond to the following groups of accessions: (1) Sabah cultivars plus many of the Sabah weed accessions, along with *indica* rice landraces from Sabah and neighboring countries (yellow); (2) Peninsular Malaysia cultivars and their Peninsular Malaysia weedy descendants (green); (3) many Peninsular Malaysia weeds plus some Sabah weeds and a few wild rice accessions (purple); (4) *tropical japonica* rice varieties, including most landraces from Sabah and neighboring countries, along with US crop varieties and two Sabah weed accessions (blue); and (5) most wild rice accessions (red).

The predominance of the yellow genetic component in Sabah high-yielding cultivars (SbCV) and Indonesian Kalimantan (KN) landraces points to these Bornean *indica* cultivated rice varieties as candidate progenitors of many of the local Sabah weed strains (Figure 2). In contrast, the near absence in Sabah weeds of the blue *tropical japonica* component that characterizes most Sabah landraces suggests that local landraces are not the major source of the recent weedy rice outbreak in East Malaysia. Also conspicuously absent from the Sabah weeds is the genetic component characteristic of Peninsular Malaysia modern elite cultivars and their weedy derivatives (green). This suggests that the elite cultivar-derived weedy rice that is so prevalent in Peninsular Malaysia has not become established in East Malaysia. Besides the predominant (yellow) population component, some Sabah weeds are characterized by a component that is most common in brown- and black hull Peninsular Malaysia weeds (purple). The presence of this genetic component suggests weed introductions from the peninsular mainland; if that is the case, however, it is not immediately apparent why Peninsular Malaysia weeds with the ‘purple’ genetic subgroup became established in Sabah and while the more common ‘green’ elite cultivar-derived Peninsular Malaysia weeds have not (see discussion below). The ‘purple’ component is also characteristic of some wild rice accessions; since wild rice is not present in East Malaysia, any Sabah weedy rice ancestry from wild rice would necessarily have to be indirect.

In the PCoA, over half of the total genetic variation in the samples could be described within the first two coordinates (59.2% without Peninsular Malaysia weeds, Figure S3A; 54.7% with Peninsular Malaysia weeds included, Figure S3B). Groupings are congruent with the STRUCTURE results at K = 5, with the Sabah weed accessions broadly clustering with Sabah cultivars, some *indica* landraces from neighboring regions, and some Peninsular Malaysia weeds. Consistent with quantifications of within-group genetic diversity (Table 1), the Sabah cultivars were less tightly clustered than the Peninsular Malaysia elite cultivars.

### An-1 sequence variation

A total of ten polymorphisms were identified in the 566-bp region spanning *An-1* exons 1 and 2. These SNPs were grouped into ten haplotypes (H1-H10; Table S6, Supporting information) among different rice groups. Consistent with sequence polymorphisms identified by Luo *et al.* (2013), we found that *An-1* haplotypes were shared between awnless and awned rice accessions (Table S6, Supporting information), suggesting that other genetic polymorphisms beyond those in *An-1* affect awn development in rice. Both awned and awnless Sabah weeds carried four haplotypes (H1, H2, H3, and H4) (Table 2; Table S3, Supporting information). The majority of Sabah weeds that are characterized by the yellow population component in the STRUCTURE analysis (Figure 2) carry H1 (76%); this haplotype is also the majority haplotype in all cultivated rice varieties with the yellow population component (Sabah cultivars and other *indica* rice varieties from neighboring regions). For the Sabah weeds characterized by the purple population component in the STRUCTURE analysis, H3 was the most common haplotype (79% of accessions). H3 is also the highest-frequency haplotype in Peninsular Malaysia weedy rice with the purple population component (present at 41% frequency), whereas it was not detected in any other rice group. This haplotype distribution supports our hypothesis of a Peninsular Malaysian origin of the Sabah weedy rice strains with the ‘purple’ genetic component. Peninsular Malaysia weed accessions also carry the other three haplotypes observed in Sabah weeds (H1, H2, H4), lending further support to the conclusion that they are a likely contributor to the Sabah weed populations (See also haplotype network, Supplementary Figure S4). Thus, distributions of the *An-1* haplotypes among the genotyped accessions are consistent with STRUCTURE results in pointing to brown-hull Peninsular Malaysia weeds (*i.e.*, those characterized by the purple population component in the STRUCTURE output), as well as Sabah cultivars and other *indica* varieties from neighboring countries (*i.e.*, those characterized by the yellow genetic component), as genetic sources of the Sabah weed strains.

**Table 2:** *An-1* haplotypes present in Sabah weeds and their distributions in potential source populations. Subgroups are defined by color corresponding to the STRUCTURE output in Figure 2 (>70% membership assignment)

Haplotype	Sabah weedy (yellow; n = 29)	Sabah weedy (purple; n = 19)	Sabah cultivars (yellow; n = 5)	Sabah Landraces (yellow; n = 3)	SE Asian Landraces (yellow; n = 13)	KN Landraces (yellow; n = 7)	PM weedy (purple; n = 37)
H1	22 (75.9%)	4 (21.1%)	5 (100.0%)	3 (100.0%)	10 (76.9%)	7 (100.0%)	12 (32.4%)
H2	2 (6.9%)	0	0	0	0	0	1 (2.7%)
H3	3 (10.3%)	15 (78.9%)	0	0	0	0	15 (40.5%)
H4	2 (6.9%)	0	0	0	1 (7.7%)	0	6 (16.2%)

## Discussion

The rapid proliferation of weedy rice in Asia and other world regions in recent decades has generated growing interest in the origins of weed infestations and the mechanisms by which weedy rice adapts as it spreads (Gross *et al.* 2010; Huang *et al.* 2017; Li *et al.* 2017; Pusadee *et al.* 2013; Song *et al.* 2014; Vigueira *et al.* 2019; Wedger *et al.* 2019). The present study examined weedy rice populations in Sabah, East Malaysia, on the island of Borneo, where the weed infestation emerged about a decade after the first report of weedy rice in the peninsular mainland of Malaysia (Wahab and Suhaimi 1991). In marked contrast to the mainland weed infestation, where modern elite ‘MR’ Malaysian cultivars were found to play a major role in the evolution of weed populations (Song *et al.* 2014), we find no evidence that Peninsular Malaysia elite cultivars have contributed to Sabah weedy rice evolution. However, other Peninsular Malaysia weedy rice ecotypes show close genetic similarity to the Sabah weeds, both in genome-wide SSR markers and *An-1* haplotypes, particularly those characterized by darker-pigmented hulls; this suggests at least some role for Peninsular Malaysia weeds in the more recent weedy rice infestation. In addition to this subset of Peninsular Malaysia weeds, other contributors to the Sabah weed populations may include local Sabah cultivars and/or other *indica* rice varieties cultivated elsewhere in Borneo (possibly Kalimantan, Indonesia) or in other neighboring countries. However, our data do not allow us to pinpoint the location and origin of the cultivars. Below we discuss these inferences and their implications for understanding the mechanisms by which weedy rice is evolving in Southeast Asia.

### Selective establishment of Peninsular Malaysian weedy rice on Borneo?

Both the SSR marker analyses and *An-1* haplotype distributions suggest that a subset of the weedy rice ecotypes found on the continental mainland have become established on Borneo. Based on the SSR dataset, approximately 16% of the Sabah weed accessions (22 out of 138) are characterized by the purple genetic component (inferred ancestry membership >70%) identified in the STRUCTURE output and correspond to Peninsular Malaysia weeds characterized by brown-pigmented hulls (PMBR accessions). It is notable that with a very extensive sample size (564 accessions representing weedy, cultivated and wild rice), the only accessions besides these Peninsular Malaysia weeds that were characterized by the purple genetic component were a small number of Peninsular Malaysia wild rice accessions; these have previously been inferred to have played a role in the origin of Peninsular Malaysia weedy rice (Cui *et al.* 2016; Song *et al.* 2014), and they also carry the relatively rare H4 *An-1* haplotype that is present in some Peninsular Malaysia and Sabah weed accessions. Thus, as there are no wild rice populations in East Malaysia, wild rice can most likely be eliminated as a direct progenitor of the Sabah weeds, and is instead probably an indirect progenitor via its role in the evolution of Peninsular Malaysia weed populations. While Peninsular Malaysia and East Malaysia are geographically separated by the South China sea, and while rice farming practices differ between the two regions — including rice variety preferences — the lack of an international boundary would facilitate informal seed sharing and accidental weedy rice introductions via contaminated seed stocks. Accidental introductions of weedy rice in seed stocks is a common mechanism for the spread of the weed in many world regions (Imaizumi 2018; Londo and Schaal 2007; Pusadee *et al.* 2013; Reagon *et al.* 2010).

If Peninsular Malaysia weedy rice populations are indeed the source of some of the Sabah weed strains, the question then arises as to why the other major genetic component in Peninsular Malaysia weeds — *i.e.*, the modern elite cultivar-derived ‘green’ component — is altogether absent in East Malaysia. We propose two possibilities. One is that the Sabah weedy rice populations became established prior to the widespread proliferation of the elite cultivar-derived weeds on the peninsular mainland. Under this scenario, brown-hull weedy rice strains were introduced into Sabah from Peninsular Malaysia two or more decades ago and only emerged as major weeds more recently; this emergence could plausibly coincide with increasing use of the mechanized direct-seeding and no-till farming practices associated with the proliferation of weedy rice (Chauhan 2013). Alternatively, Peninsular Malaysia cultivar-derived weeds could have been introduced but failed to establish. This might reflect chance demographic effects and/or out-competition by weed strains better adapted to local growing conditions. In this regard, it is notable that the Peninsular Malaysian elite ‘MR’ cultivars that were the source of these Peninsular Malaysia weeds have not been widely adopted by rice farmers in Sabah. If soil or other conditions in Sabah are not conducive for the successful growing of these cultivars, this could conceivably account for a lack of proliferation of their weedy descendants. Direct assessments of ‘MR’ elite cultivar performance in Sabah rice fields could be used to further explore this hypothesis.

### Additional sources of Sabah weedy rice

The predominant genetic component in Sabah weedy rice is almost entirely absent from cultivated and weedy rice in Peninsular Malaysia, which suggests that the mainland is not the source of these weed strains. While genetic resolution of our SSR and *An-1* analyses does not allow for a definitive inference on the source of these weeds, two candidates appear most likely: Sabah high-yielding cultivars (SbCV), and *indica* landraces from Sabah or nearby regions. Between these alternatives, the former may be the more likely candidate. Whereas rice landraces have long been a component of Malaysian rice production (albeit with declining importance in recent decades), Sabah cultivars represent newly-introduced genetic material whose widespread cultivation proceeds weedy rice outbreaks by a relatively few years. In addition, most landraces in East Malaysia are *tropical japonica* varieties and could not be the source of these Sabah weeds. Sabah cultivars are also characterized by a level of genetic diversity that is similar to that of Sabah weeds. All of these factors point to the widely-cultivated Sabah cultivars as strong candidates to be progenitors of the Sabah weeds. If true, this pattern would indicate that the transition to commercialized rice farming in Malaysia has had the unintended consequence of creating two independently-evolved, cultivar-derived weedy rice ecotypes. This should be considered, at minimum, a cautionary tale for other Asian countries that are now transitioning to mechanized rice production. However, definitive determination of whether these Sabah weeds are descendants of Sabah cultivars must await analysis using higher-resolution genetic markers, such GBS-derived SNPs, which are now being applied in studies of Asian weedy rice evolution (Huang *et al.* 2017; Vigueira *et al.* 2019). With the availability of these high-density SNP data, we are able to then perform explicit model-based testing for goodness of fit of the STRUCTURE results which could help to rule out the possibility that inferences on group admixture are artifacts of demographic history or unsampled source populations (Lawson *et al.* 2018).

### Enriched genetic diversity in Southeast Asian weedy rice

Considering the small sampling area studied in East Malaysia (approximately 250 km^2^), the higher genetic diversity estimated for Sabah weedy rice populations (*H*_e_ = 0.46) than in many previously studied weedy rice populations [Cao *et al.* (2006), *H*_e_ = 0.31; Gealy *et al.* (2009), *H*_e_ = 0.27; Song *et al.* (2014), *H*_e_ = 0.37] is noteworthy. The cultivars of Sabah are characterized by high genetic diversity (*H*_e_ = 0.48), which suggests that these candidate progenitors of the Sabah weeds could be contributors of this high genetic diversity. Consistent with our findings, Jiang *et al.* (2012) and Pusadee *et al.* (2013) have similarly reported positive correlations for high genetic diversity in cultivars and co-occurring weed populations. Findings of the present study are thus potentially in line with a general phenomenon of higher genetic diversity in weedy rice populations where crop-to-weed gene flow occurs with some frequency, as reported by Chen *et al.* (2004) for weed populations in China and Korea and by Pusadee *et al.* (2013) for Thai weedy rice. Such on-going crop-weed hybridizations and introgressions would enhance adaptation, including crop mimicry, among weedy rice strains in fields (Mispan *et al.* 2013; Xia *et al.* 2011), and ultimately increase the overall genetic diversity of weedy rice populations in these regions.

Specific cases of hybridization between cultivars and Sabah weedy rice types can be inferred in some cases from the combined results of our STRUCTURE and PCoA analyses. For example, weedy rice accessions SHA-SBBG25 (awned), BR-SBTA22 (opened-panicle), BR-SBTA06 (opened-panicle) and BR-SBNT05 (short grain), which are located within the loose assemblage of accessions between Sabah cultivars and the Sabah brown-stripe-hull awnless accessions, and share inferred ancestry values of approximately 0.5 for both Sabah cultivar and Sabah brown-stripe-hull awnless-like genotypes. Further scrutiny of these accessions reveals intermediate morphological characters as evidence of hybridization between cultivars and weedy rice types (*e.g.*, white pericarp, low seed shattering, and semi-open-panicle structure). As synchronization of heading time between cultivars and weedy rice is commonly observed in Sabah rice fields, this may have caused complex hybridization events among weeds and cultivar strains, and eventually produced a widely variable array of weedy rice genotypes (Fujino *et al.* 2010; Langevin *et al.* 1990). Such admixture between Sabah cultivar (or possibly Kalimantan, KN or SbLr landraces) and Sabah brown-stripe-hull awnless -like weeds may have promoted further hybridization between Sabah weedy rice and cultivars groups, potentially contributing to the further formation of new weedy rice types (Londo and Schaal 2007).

Exchange and sharing of self-supplied rice seeds among farmer communities have long been seen as part of the agricultural traditions in Southeast Asia (Pusadee *et al.* 2013). These practices, along with the shared use of combine harvesters across planting areas, are important factors promoting weedy rice proliferation (Barroso *et al.* 2006; Calha *et al.* 2014; Karim *et al.* 2004). In Malaysia, uncertified crop seeds are regularly contaminated by weedy rice, promoting the widespread occurrence of weedy rice in the country (Song *et al.* 2014). In comparison to Peninsular Malaysia, weeds in Sabah would further benefit from the greater standing variation present in Sabah cultivars compared to the elite MR cultivars widely adopted on the mainland (see Table 1). This genetically enriched background would allow Sabah weedy rice to rapidly adapt as an agricultural weed. Similarly, Kanapeckas *et al.* (2018) suggest that adaptive combinations of traits enable California weedy rice populations to survive modern agriculture practices with strong selection pressures and spread rapidly. An initial lack of awareness of the severity of the weedy rice outbreak in East Malaysia likely further negatively impacted weed control management and further intensified adaptation and wide-spread of weedy rice across rice growing regions in Sabah.

## Conclusion

Our SSR and *An-1* sequence analyses support the conclusion that the genetic composition and evolution of Sabah weedy rice has been shaped by accidentally-introduced Peninsular Malaysia weedy rice strains. Recent weed genetic studies suggested that introgression with local cultivars and natural adaptation together shaped the evolution of weedy rice in China (Sun *et al.* 2012; Xia *et al.* 2011). Besides *An-1*, it will be interesting to examine genetic variation at some other domestication-related genes in Sabah weed populations, to clarify the role of cultivar-to-weed introgressions associated with weedy rice adaptation.
